# Professional Aphorisms

**Published:** 1853-01

**Authors:** 


From the Ohio Medical and Surgical Journal.

*Professional Aphorisms.*

The talented editor of *L' Union Medicale*, lately gave a few extremely apposite and amusing professional aphorisms, in one of his clever *fuilletons*. We shall just extract a few:

1. Life is short, the making of a practice difficult, and professional brotherhood deceptive. 2. A man's practice may be compared to a field. on which *tact* acts as a manure. 3. A medical practice may be likened to a flannel waistcoat—neither can be left one moment without risk. 4. The practitioner who is often absent runs the same danger as the lover, for both may find themselves supplanted on their return. 5. Take great care of your first patients, ye beginners, for these are the seed from which your practice is to spring. 6. When a medical man wishes to get rid of a troublesome patient he need but send in his bill. 7. The practitioner who expects his reward from the gratitude of his patients, may be like the countryman who waited, in order to cross the river, until the waters had done flowing. 8. To ask an exorbitant fee always redounds to the disgrace of the profession. A wealthy patient who was asked an enormous sum by a surgeon, after an operation, answered, "You ought to have said at first, your money or your life." 9. When the blind credulity of the public in medical matters is considered, one does not wonder that there are so many quacks and impostors, but on the contrary, that there are still so many upright medical men. 10. Consultations are either very useful or very dangerous, just as the medical attendant knows how to manage. It is foolish to have recourse to them too often, and still more foolish to reject them altogether. Don't wait till the friends of the patient ask for a consultation; but don't talk of a consultation if you think the result will be favorable. 11. It is not an easy task to come out of a consultation without being a little lowered in the estimation of the patient and his friends,—the more so as there are physicians and surgeons who with the utmost urbanity, throw out perfidiously, concealed hints, which the practitioner should immediately take up, and boldly insist upon a clear statement. 12. A consultation is often a note of hand drawn by the usual attendant upon the patient, for the benefit of the physician called in to give his opinion.

